# Gut microbiome remodeling in chronic kidney disease: implications of kidney replacement therapies and therapeutic interventions

**DOI:** 10.3389/fmed.2025.1620247

**Published:** 2025-07-15

**Authors:** Qianwei Wang, Yucheng Han, Liang Pang, Zhicheng Zhou, Lijuan Dai

**Affiliations:** ^1^Graduate School, Heilongjiang University of Chinese Medicine, Harbin, China; ^2^Graduate School, Zhejiang University of Chinese Medicine, Wenzhou, China; ^3^Graduate School, Anhui University of Chinese Medicine, Hefei, China; ^4^Department of Nephrology, First Affiliated Hospital of Heilongjiang University of Chinese Medicine, Harbin, China

**Keywords:** end-stage renal disease, gut microbial metabolites, plant-based diet, probiotics, fecal microbiota transplantation

## Abstract

The escalating global burden of end-stage renal disease (ESRD), driven by aging populations and rising metabolic comorbidities, underscores the urgent need for innovative therapeutic strategies. Emerging evidence highlights the gut microbiome as a pivotal modulator of renal pathophysiology through the gut-kidney axis, with microbial dysbiosis exacerbating gut microbial metabolites (e.g., uremic toxins), systemic inflammation, and multi-organ damage. This narrative review explores the divergent impacts of kidney replacement therapies (KRT)—hemodialysis (HD) and peritoneal dialysis (PD)—on gut microbiota dynamics: HD is associated with Firmicutes and Proteobacteria enrichment, reduced butyrate-producing taxa (e.g., *Faecalibacterium*, *Roseburia*), and systemic microbial translocation; whereas PD-driven glucose absorption and iron supplementation foster pathogenic proliferation (e.g., *Enterobacteriaceae*) and impair short-chain fatty acid (SCFA) metabolism. Current interventions, including probiotics, prebiotics, plant-based diets (PBDs), and fecal microbiota transplantation (FMT), demonstrate potential in mitigating dysbiosis and uremic toxin accumulation. PBDs reduce inflammatory markers (IL-6, CRP) and lower all-cause mortality risk by 24% in PD patients; synbiotics (e.g., *Lactobacillus casei* + galactooligosaccharides) reduce serum p-cresyl sulfate by 20% in HD patients; and FMT increases levels of short-chain fatty acids (propionate, butyrate) and lowers trimethylamine N-oxide (TMAO) concentrations in streptozotocin-induced diabetic nephropathy mouse models. However, clinical translation remains challenged by small sample sizes, heterogeneous outcomes, and a lack of hard endpoints. Future research must prioritize standardized protocols, personalized microbial profiling, and synergistic integration of dietary and microbiome-targeted therapies. Bridging mechanistic insights with clinical validation will advance precision medicine in ESRD management, offering transformative potential for patients burdened by this therapeutic impasse.

## 1 Introduction

The 2019 Global Kidney Health Atlas (GKHA) cross-sectional epidemiological survey reveals an escalating global burden of end-stage renal disease (ESRD), with an annual incidence of 144 cases per million population (1). This trajectory is exacerbated by demographic aging and the mounting prevalence of diabetes, hypertension, and obesity. ESRD not only compromises patients’ quality of life but imposes staggering socioeconomic burdens—exemplified by annual Medicare expenditures exceeding $49.2 billion for ESRD-related care in the United States, a financial strain that has persistently intensified over the past decade (2).

While kidney replacement therapy (KRT)—encompassing hemodialysis (HD), peritoneal dialysis (PD), and renal transplantation—has prolonged survival, ESRD continues to markedly truncate life expectancy. Five-year survival rates post-ESRD onset remain suboptimal at 41.4% for HD and 46.9% for PD patients (2). HD necessitates vascular access via arteriovenous fistulas or central venous catheters, coupled with rigorous dietary constraints, and remains the predominant modality, particularly in resource-limited settings (3). PD, despite its advantages in home-based convenience and residual renal function preservation, is intrinsically limited by refractory complications including peritonitis, uremic toxin accumulation, and chronic low-grade inflammation (4). Renal transplantation, though the gold standard for ESRD, remains inaccessible to most patients due to critical donor shortages (5). This therapeutic impasse has catalyzed a paradigm shift toward investigating systemic regulation of the gut-kidney metabolic axis, transcending conventional neuropathological frameworks.

Emerging evidence suggests that the gut microbiota, as a key component of the human “meta-organ,” serves as a critical regulator of renal pathophysiology (6, 7). Patients with end-stage renal disease (ESRD) exhibit severe microbial dysbiosis: opportunistic pathogens (e.g., *Staphylococcus*, *Enterococcus*, *Helicobacter*) show significantly increased abundance in the gut, while short-chain fatty acid (SCFA)-producing bacteria with renoprotective effects (e.g., *Faecalibacterium*, *Lachnospira*) are significantly reduced (8, 9). Among these, pathogen-derived uremic toxins (e.g., indoxyl sulfate, p-cresol) disrupt the integrity of the intestinal barrier (“intestinal leakage”), trigger systemic inflammatory cascades, and accelerate renal deterioration (10). Notably, different KRT modalities exhibit marked heterogeneity in reshaping gut microbial ecology: PD patients, due to prolonged exposure to high-glucose dialysate, demonstrate fundamentally distinct microbiota profiles compared to HD patients, while renal transplant recipients under immunosuppressive states may experience exacerbated microbial dysbiosis (11, 12). These modality-dependent microbial signatures unveil novel targets for precision interventions.

This review pioneers a systematic dissection of KRT-driven gut microbiome remodeling patterns in ESRD. Through the lens of gut-kidney crosstalk mechanisms, we critically evaluate therapeutic boundaries of probiotics, prebiotics, dietary fiber, and fecal microbiota transplantation (FMT), proposing a translational roadmap for personalized ESRD management.

## .2 Methods

To establish the evidence base for this review, we systematically searched three core databases: PubMed, Web of Science, and Embase, supplemented by manual searches in Google Scholar to capture gray literature. The search strategy employed a combination of Medical Subject Headings (MeSH terms) and free-text terms, with a focus on “end-stage kidney disease (ESKD),” “gut microbiome,” “kidney replacement therapy (KRT),” “hemodialysis (HD),” “peritoneal dialysis (PD),” “gut-targeted interventions (probiotics/prebiotics/synbiotics/plant-based diets/FMT),” “uremic toxins,” and “short-chain fatty acids (SCFA),” ensuring coverage of multi-dimensional associations among gut microbiome remodeling, KRT effects, and intervention strategies in ESKD.

The search logic was optimized using the PICO framework (Population: ESKD patients; Intervention: HD/PD treatment, probiotics/prebiotics/plant-based diets/FMT; Comparison: differences between KRT modalities or intervention types; Outcome: gut microbiota composition/function, SCFA metabolism, uremic toxin levels, inflammatory markers, and clinical outcomes) to precisely extract target studies.

Given the rapid advancements in gut microbiome research, the literature search was limited to the period 2014–2024 to focus on the latest mechanistic explorations and clinical translational evidence in this field. Inclusion criteria included: ➀ clinical interventional studies [e.g., randomized controlled trials (RCTs), cohort studies, cross-sectional studies]; ➁ animal models (e.g., adenine-induced CKD models, streptozotocin-induced diabetic nephropathy models); and ➂ mechanistic studies (e.g., microbiome sequencing).

## 3 The gut microbiome

### 3.1 Importance of the gut microbiota and its metabolites

Contemporary scientific discourse has elevated the gut microbiome to the status of a quasi-autonomous organ, with emerging evidence demonstrating its metabolic capacity surpasses hepatic functionality in orchestrating host metabolic regulation, immune homeostasis, and systemic physiology (13, 14). This intricate ecosystem derives its biological significance from staggering biodiversity—the human gut harbors over 100 trillion microbial cells encompassing ≥ 35,000 species, predominantly composed of Firmicutes and *Bacteroidetes* phyla, which together with *Proteobacteria* and *Actinobacteria* constitute a dynamically balanced microbial community (15, 16).

Within the approximately 5–7 m-long intestinal tract, microbial communities interact with the host environment to form highly differentiated ecological niches. The small intestine, characterized by its acidic conditions, rapid food transit, and antimicrobial molecule restrictions, is predominantly colonized by facultative anaerobes. In contrast, the colon, with its anaerobic environment and abundant undigested dietary residues, serves as the primary habitat for complex microbial populations such as *Prevotellaceae*, *Rikenellaceae*, and *Lachnospiraceae* (17). However, this spatial distribution is not static—factors including dietary patterns, medication use, and intestinal motility frequency can dynamically reshape microbial composition (18).

Microbial-host interactions extend beyond structural characteristics to exert broad physiological effects through metabolic activities. In nutrient metabolism, gut microbiota break down dietary fibers and complex polysaccharides to generate short-chain fatty acids (SCFAs) such as acetate, propionate, and butyrate (19). These metabolites not only serve as energy sources for the host but also regulate metabolic balance and anti-inflammatory responses (20). Notably, experiments by Lanza et al. demonstrated that orally administered SCFAs act as significant modulators of neuroinflammation in migraine pathology and potent regulators of perivascular neural fiber activation in the gut system (21). Additionally, gut microbiota participate in bile acid transformation and recycling, as well as the synthesis of nutrients like folate, vitamin K, and vitamin B12 (22). For instance, *Prevotella* and *Ruminococcus* dominate folate and vitamin K/B12 production, while *Bifidobacterium* and *Lactobacillus* function as primary producers of folate and B12, respectively. Microbial deconjugation of conjugated bile acids occurs through hydroxyl group oxidation at C-3, C-7, and C-12 positions, forming oxidized bile acids, which are subsequently reduced to α- or β-configurations (23–26).

Beyond their role in metabolic support, gut microbiota play a critical role in regulating the host immune system (27). They recognize microbial molecular patterns through pattern recognition receptors [e.g., Toll-like receptors (TLRs), NOD-like receptors (NLRs)], activate immune signaling pathways, and dynamically balance inflammatory responses with immune tolerance (28, 29). Meanwhile, a healthy microbial equilibrium enhances intestinal epithelial barrier function to defend against pathogen invasion by promoting the expression and stabilization of tight junction proteins and regulating mucus properties. Peterson et al. observed that germ-free mice exhibit extremely thin colonic mucus layers, but exposure to bacterial products (peptidoglycan or lipopolysaccharide) rapidly restores mucus thickness to levels seen in conventionally raised mice (30).

### 3.2 Gut dysbiosis: a catalyst for systemic pathogenesis

Gut microbiota dysbiosis, characterized by reduced diversity, an abnormal Firmicutes/*Bacteroidetes* ratio, and excessive proliferation of pathogenic bacteria, is closely associated with various diseases.

Dysbiosis leads to reductions in beneficial metabolites [e.g., short-chain fatty acids (SCFAs)] and promotes the production of pathogenic metabolites [e.g., indoxyl sulfate (IS), p-cresyl sulfate (pCS), trimethylamine N-oxide (TMAO)] (31, 32). In patients with chronic kidney disease (CKD), uremia and reduced renal clearance lead to elevated gastrointestinal urea levels, which are hydrolyzed into ammonia by bacterial ureases. This alkaline shift suppresses SCFA-producing commensal bacteria while promoting excessive proliferation of proteolytic pathogens (e.g., *Enterobacteriaceae*, *Clostridium*) capable of producing uremic toxins (indoxyl sulfate, p-cresyl sulfate) (33). These toxins impair intestinal barrier function, allowing endotoxins [e.g., lipopolysaccharide (LPS)] to translocate into systemic circulation. Once absorbed, they activate Toll-like receptor 4 (TLR4) and nuclear factor kappa-B (NF-κB) pathways, exacerbating chronic inflammation and oxidative stress—major contributors to renal fibrosis and cardiovascular complications (34, 35).

The impact of gut dysbiosis extends beyond the kidneys to systemic organs. Trimethylamine N-oxide (TMAO), a metabolite of dietary choline, promotes macrophage foam cell formation and platelet aggregation, thereby exacerbating atherosclerosis (36, 37). Similarly, p-cresyl sulfate (pCS) disrupts the integrity of the blood-brain barrier via epidermal growth factor receptor (EGFR)/STAT3 signaling, linking gut-derived toxins to cerebrovascular risk in CKD patients (38).

Furthermore, with the deepening of research, gut dysbiosis has been found to be associated with various gastrointestinal diseases (39), metabolic disorders (e.g., obesity, insulin resistance, type 2 diabetes) (40), and even neurological disorders (e.g., Alzheimer’s disease, Parkinson’s disease) (41).

In summary, gut microbiota are not merely passive participants in human physiological activities but act as critical drivers of disease pathogenesis. Therefore, systematically elucidating the core mechanisms of gut microbiota in renal function is crucial for providing new insights to break through traditional renal pathophysiological frameworks.

## 4 Core mechanisms of the gut-kidney axis

### 4.1 Microbial-derived uremic toxin metabolism

The gut microbiome serves as the principal bioreactor for uremic toxin generation, with its metabolic byproducts directly interfacing with renal pathophysiology through the gut-kidney axis. Characterized toxins of microbial origin include indoxyl sulfate (IS), p-cresyl sulfate (PCS), phenylacetylglutamine, and trimethylamine N-oxide (TMAO) (31, 42). While biosynthetically distinct, these compounds collectively orchestrate a pathogenic network through convergent mechanisms of renal and systemic toxicity.

#### 4.1.1 Aromatic amino acid metabolism and toxin generation

Indoxyl sulfate (IS) originates from microbial degradation of tryptophan in the colon. Tryptophan is metabolized by *Escherichia coli* and other proteolytic bacteria into indole, which undergoes hepatic hydroxylation and sulfation to form IS (32). IS exhibits high albumin-binding affinity, and its clearance via hemodialysis is restricted to the unbound fraction due to protein-binding limitations (43). Consequently, IS accumulates and induces endothelial oxidative stress and renal fibrosis through activation of the aryl hydrocarbon receptor (AhR)/nuclear factor kappa-B (NF-κB) pathway (44).

Aryl hydrocarbon receptor, as a ligand-activated transcription factor, relies on dual regulation by both endogenous and exogenous ligands for its function. Exogenous ligands primarily originate from the metabolism of environmental pollutants. For example, *1-methoxypyrene* (a metabolite of polycyclic aromatic hydrocarbons) can directly enter the circulatory system and bind with high affinity to the PAS-B domain of AhR (45, 46).

Among endogenous ligands, indoxyl sulfate (IS) serves as a core activator of AhR in chronic kidney disease (CKD): IS is actively transported into the cytoplasm via organic anion transporters (e.g., OAT1/3) on the membrane of proximal tubular epithelial cells (38). Upon entering the cell, IS binds to cytosolic AhR, triggering its conformational changes and release of chaperone proteins (e.g., HSP90), exposing the nuclear localization signal (NLS), and promoting AhR translocation into the nucleus (38). Within the nucleus, AhR forms a heterodimer with ARNT, directly binds to the XRE element (5′-GCGTG-3′) in the promoter regions of profibrotic genes (e.g., TGF-β1, Cyp1a1), and activates the transcription of target genes (47). This process significantly upregulates the expression of α-smooth muscle actin (α-SMA) and collagen type I (Col-I), induces epithelial-mesenchymal transition (EMT) of renal tubules, and ultimately exacerbates renal interstitial fibrosis (48, 49).

Activated AhR amplifies renal injury through dual pathological mechanisms: First, it forms a dynamic regulatory network with the NF-κB pathway—early IS-activated AhR binds to the p65 subunit of NF-κB, leading to mutual inhibition of both in the cytoplasm and limiting the initial release of pro-inflammatory factors; subsequently, AhR induces the expression of suppressor of cytokine signaling 2 (Socs2), relieving the inhibition of NF-κB, and ultimately, AhR directly binds to the TNF-α promoter region, driving the transcription of pro-inflammatory factors such as IL-6 and TNF-α, thereby exacerbating systemic micro-inflammation (44). Second, AhR mediates the ubiquitination and degradation of peroxisome proliferator-activated receptor γ coactivator 1-α (PGC1α) via the ubiquitin-proteasome system (47). As a core regulator of mitochondrial biogenesis, the loss of PGC1α leads to impaired mitochondrial DNA (mtDNA) replication, decreased activity of respiratory chain complexes (e.g., complexes I-IV), and reduced ATP production, ultimately inducing senescence and functional loss of renal tubular cells (44).

Aromatic amino acids tyrosine and phenylalanine undergo bacterial deamination, transamination, and decarboxylation in the distal colon to form phenolic compounds such as phenol and p-cresol (50). The majority of intestinal p-cresol is absorbed into systemic circulation, while a residual fraction undergoes glucuronidation by host epithelial UDP-glucuronosyltransferases to form p-cresyl glucuronide. Systemic p-cresol is sulfated in the liver to produce p-cresyl sulfate (pCS) (51). The free fraction of these compounds undergoes glomerular filtration, while conjugated forms are secreted via tubular epithelial cells, both ultimately excreted in urine (52, 53). The pathological impacts of pCS are increasingly elucidated:

A 2015 CKD rat model demonstrated pCS-induced overproduction of reactive oxygen species (ROS), activation of nicotinamide adenine dinucleotide phosphate (NADPH) oxidase, and increased caspase-3 activity, promoting cardiomyocyte apoptosis (54).

A meta-analysis by Lin et al. (*n* = 1,572) confirmed significant associations between pCS and all-cause mortality/cardiovascular events in CKD patients (55).

Animal models by Sun et al. revealed that pCS-mediated activation of the renin-angiotensin system (RAS)/transforming growth factor-β (TGF-β) pathway induces renal tubular epithelial-mesenchymal transition (EMT), leading to renal fibrosis (49). Recent studies identified circulating pCS levels as a risk factor for cerebrovascular events in CKD patients. pCS activates epidermal growth factor receptor (EGFR) signaling, sequentially triggering intracellular annexin A1 and signal transducer and activator of transcription 3 (STAT3), which mobilize matrix metalloproteinases (MMP-2/9) and disrupt blood-brain barrier integrity (38).

#### 4.1.2 Choline metabolism and the controversial role of TMAO

Distinct from other uremic toxins, trimethylamine N-oxide (TMAO) exhibits unique metabolic characteristics and pathological effects. Choline, phosphatidylcholine, and L-carnitine are metabolized by gut microbiota (e.g., Proteobacteria) into trimethylamine (TMA), which is absorbed and oxidized by hepatic flavin monooxygenase to form TMAO (56). Unlike protein-bound toxins like indoxyl sulfate and p-cresyl sulfate, TMAO can be effectively removed via dialysis. However, its residual toxicity remains clinically significant. Studies demonstrate that TMAO exacerbates atherosclerosis and thrombosis by promoting platelet Ca^2^ + release (36) and upregulating macrophage CD36/scavenger receptor-A1 (SR-A1) expression (37). Although its pathogenic mechanisms remain incompletely understood, TMAO is widely recognized as an early biomarker for cardiovascular risk in CKD patients (57).

### 4.2 Drivers of gut dysbiosis and intestinal barrier dysfunction

#### 4.2.1 Urea-ammonia cycle: the initiation of microbial imbalance

Urea, a primary metabolic waste product of the kidneys, is hydrolyzed by bacterial ureases into ammonia and carbamate. Ammonia is further converted to ammonium hydroxide, elevating intestinal pH and compromising epithelial barrier integrity (58, 59). This eroded barrier triggers leukocyte infiltration, local inflammation, and proinflammatory cytokine release (TNF-α, IFN-γ, IL-1β, IL-12), which disrupt tight junction integrity and increase barrier permeability (59, 60).

In CKD patients, heightened gastrointestinal urea secretion creates a dysbiotic environment that suppresses beneficial bacteria (*Lactobacillus*, *Bifidobacterium*) while promoting pathogenic colonization (*Enterococcus*, Proteobacteria) (33, 61). Over 50% of gut microbiota in these patients encode urease genes, accelerating ammonia and uremic toxin production. Wong et al. systematically categorized bacterial families harboring urease, uricase, p-cresol/indole-producing enzymes, and SCFA-synthesizing genes in 12 healthy controls and 24 ESRD patients. Their analysis revealed significant enrichment of urease/uricase/indole/p-cresol-producing taxa and depletion of butyrate-producing *Lachnospiraceae* in ESRD cohorts (33).

#### 4.2.2 SCFAs: bidirectional regulators of intestinal barrier homeostasis

Chronic kidney disease patients frequently experience intestinal wall congestion, edema, antibiotic overuse, metabolic acidosis, and reduced dietary fiber intake (62). Commensal bacteria (*Bifidobacterium*, *Faecalibacterium*) ferment dietary fiber to produce SCFAs (acetate, propionate, butyrate), which activate G protein-coupled receptors to enhance tight junction protein expression (ZO-1, occludin) and suppress inflammatory cytokines (63–65). However, low-fiber diets diminish SCFA production, while oral iron supplements and antibiotics exacerbate microbial imbalance. Reduced SCFA-producing taxa (*Bifidobacterium*, *Lachnospiraceae*) in CKD patients increase intestinal permeability, enabling endotoxin (e.g., monocyte-derived LPS) and bacterial DNA translocation into systemic circulation. These factors activate TLR4/TLR9 signaling, triggering NF-κB/NLRP3 inflammasome pathways and releasing proinflammatory cytokines (IL-1β, IL-6), which exacerbate systemic microinflammation and organ damage (66, 67). Wang et al. confirmed using a 5/6 nephrectomy rat model that bacterial translocation induces systemic microinflammation (68). Recent breakthroughs highlight SCFAs’ extraintestinal roles:Larraufie et al. demonstrated that propionate and butyrate regulate appetite and glucose metabolism via PYY upregulation, with SCFA deficiency exacerbating obesity and diabetes (69).

Pluznick et al. revealed SCFA-mediated blood pressure regulation through Olfr78 and Gpr41 receptors, with propionate exerting receptor-dependent antihypertensive effects (70).

These findings directly link SCFAs to CKD risk factors (hypertension, diabetes), underscoring their multidimensional regulatory value (71).

The above pathomechanisms of the gut-kidney axis indicate that gut dysbiosis is not merely a consequence of ESRD but may dynamically evolve with therapeutic interventions. As the most common renal replacement therapies, hemodialysis (HD) and peritoneal dialysis (PD) exert modality-specific impacts on gut microbiota structure and function through distinct solute clearance mechanisms. These differences may hold the key to optimizing ESRD management strategies.

## 5 Impact of hemodialysis on microbiome dynamics

The interaction between hemodialysis (HD) and the gut microbiome extends beyond solute clearance, manifesting as bidirectional dysregulation of the gut-kidney axis (72). This dysregulation is first evident in phylum-level microbial alterations: studies demonstrate significant enrichment of Proteobacteria, Actinobacteria, and Firmicutes alongside reduced *Bacteroidetes* abundance in HD patients compared to healthy individuals (61, 73). Direct evidence comes from Chao et al. who analyzed fecal samples from 30 healthy controls, 15 HD patients with well-controlled calcium/phosphorus levels, and 16 hyperphosphatemic HD patients using 16S rRNA amplicon sequencing. Their findings confirmed pronounced enrichment of Firmicutes, Actinobacteria, and Proteobacteria in HD cohorts (74). Notably, microbial changes exhibit population-specific heterogeneity: Crespo-Salgado et al. reported increased Bacteroidetes abundance in pediatric ESRD patients undergoing HD via 16S rRNA sequencing (75).

Beyond changes at the phylum level, diversity indices further revealed hemodialysis (HD)-induced ecological perturbation. Studies by Wu et al. demonstrated that α-diversity indices in HD patients were similar to those in healthy individuals, whereas β-diversity analysis showed significant differences in microbiota structure between HD patients and healthy populations (73). Crespo-Salgado’s research team recently compared the gut microbiomes of pediatric patients undergoing peritoneal dialysis (PD), hemodialysis (HD), post-renal transplantation, and the healthy control group. The authors noted that the gut microbiome of HD patients was similar to that of the control group and exhibited higher diversity than that of PD patients (75).

This dysbiosis is exacerbated by depletion of key functional taxa, particularly butyrate-producing genera (*Faecalibacterium*, *Roseburia*). As butyrate serves as a critical energy source for colonocytes, its deficiency leads to colonic metabolic dysfunction and directly correlates with indoxyl sulfate accumulation and intestinal barrier impairment (64, 76).

Hemodialysis-induced microbial disruption is not confined to the gut but triggers systemic microbial translocation. In the metagenomic study by Shi et al. bacterial DNA (predominantly Firmicutes, Bacteroidetes, and Proteobacteria) was detected in the bloodstream of 27% of HD patients, and this DNA concentration positively correlated with CRP and IL-6 levels (77). The mechanism underlying this “microbe-blood interface breach” involves HD-associated uremic toxins downregulating tight junction proteins (occludin, ZO-1), thereby compromising gut-vascular barrier integrity (35).

## 6 Impact of peritoneal dialysis on gut microbiota structure and function

Peritoneal dialysis (PD), through cyclical exchanges of glucose-based dialysate for solute clearance, exerts modality-specific effects on gut microbiota composition and functionality (78). However, existing studies on PD populations remain limited and exhibit marked heterogeneity in outcomes. While some reports indicate significantly reduced α- and β-diversity in PD patients compared to healthy controls (75, 79), Gao et al. recently observed comparable α-diversity between PD patients and healthy individuals (80). Teixeira et al. further demonstrated no statistical differences in α/β-diversity between PD patients and their cohabitating household contacts (81). These contradictory findings suggest confounding influences from variables such as ESRD disease duration and comorbid conditions.

To disentangle whether gut microbiota alterations in ESRD patients arise from dialysis or uremia itself, controlled comparative studies have been conducted. Wang et al. identified distinct microbial abundance and structural differences between PD patients and non-dialyzed ESRD counterparts, particularly in ***Lactobacillus*** species (82). Conversely, Luo et al. reported no significant phylum/genus-level variations between PD and pre-dialysis cohorts (83). Despite conflicting conclusions, all studies confirm characteristic functional metabolic deviations in PD-associated microbiota compared to healthy populations.

At the phylum level, PD patients consistently exhibit microbial remodeling marked by reduced Firmicutes and Actinobacteria abundance alongside Proteobacteria enrichment (84). This dysbiotic shift may correlate with long-term oral iron supplementation—known to suppress SCFA-producing taxa (e.g., *Faecalibacterium*) and promote proteolytic bacterial proliferation (85)—coupled with systemic glucose absorption from hypertonic dialysate. Approximately 15%–20% of dialysate glucose permeates peritoneal capillaries, creating a carbon-rich milieu that favors bacterial overgrowth (86). Of particular concern is *Enterobacteriaceae* expansion, as its secreted lipopolysaccharide (LPS) activates TLR4/NF-κB signaling, directly correlating with increased Gram-negative peritonitis incidence in PD patients (87). Milan Manani et al. provided clinical evidence linking elevated dialysate LPS levels to peritonitis risk, with dialysate leukocyte counts proportionally rising alongside LPS concentrations (87).

Metabolomic analyses further elucidate core functional deficits in PD-associated microbiota. PD patients show significant reductions in fecal butyrate (BA) and caprylate (CA) levels due to depleted SCFA-producing genera (*Faecalibacterium*, *Roseburia*) (88). As previously discussed, SCFA deficiency impairs intestinal barrier integrity via downregulation of tight junction proteins (e.g., occludin) (64) and induces systemic metabolic dysregulation through disrupted energy metabolism and immunomodulatory dysfunction (12, 64).

### 6.1 Comparison of HD and PD in SCFA-mediated downstream effects

Reductions in short-chain fatty acid (SCFA)-producing bacteria (e.g., *Faecalibacterium*, *Roseburia*) in hemodialysis (HD) patients lead to downstream effects similar to those observed in peritoneal dialysis (PD) patients. SCFAs—particularly butyrate—serve as the primary energy source for colonic epithelial cells, which can promote the expression of tight junction proteins (e.g., ZO-1, occludin) via activation of G protein-coupled receptors (e.g., GPR41/43), thereby maintaining the integrity of the intestinal mucosal barrier (34, 64). In HD patients, reduced butyrate-producing bacteria result in colonic metabolic dysfunction, exacerbated intestinal barrier “leakage,” and translocation of bacterial DNA and endotoxins (e.g., lipopolysaccharide, LPS) into the circulatory system (77). PD patients similarly exhibit downregulated tight junction proteins and increased intestinal permeability due to SCFA deficiency (34, 88). Both patient groups show elevated levels of inflammatory factors such as C-reactive protein (CRP) and interleukin-6 (IL-6) in the blood (77, 87).

Moreover, disrupted intestinal barriers allow translocated microbial components (e.g., LPS, bacterial DNA) to activate TLR4/TLR9 signaling pathways, triggering NF-κB/NLRP3 inflammasome activation and the release of pro-inflammatory factors like IL-1β and IL-6 (34, 77). In HD patients, this micro-inflammation directly correlates with an increased risk of cardiovascular events (55, 89); in PD patients, peritoneal microbial translocation (e.g., overgrowth of *Enterobacteriaceae*) elevates the risk of peritonitis (87).

Notably, despite similar underlying mechanisms, HD and PD patients differ in the specific manifestations of SCFA reduction and related complications due to distinct treatment modalities. Microbial translocation in HD patients is more directly associated with blood bacterial DNA levels (77), potentially linked to uremic toxin accumulation [e.g., indoxyl sulfate (IS), p-cresyl sulfate (PCS)] induced by HD itself, which damages the gut-vascular barrier (35). In contrast, PD patients—due to glucose absorption from high-glucose dialysate and iron supplementation—are more prone to overgrowth of pathogenic bacteria like *Enterobacteriaceae* (84), with microbial translocation potentially influencing inflammation via the peritoneal-blood interface (e.g., the peritoneum) (87).

Short-chain fatty acids-producing bacterial reduction in HD patients is primarily associated with uremic status (disruption of the urea-ammonia cycle, which impairs microbial balance) and HD treatment (partial removal of beneficial metabolites) (61, 72, 73). In PD patients, this reduction is more influenced by dual factors: a high-glucose environment (promoting pathogenic bacterial proliferation) and iron supplementation (inhibiting SCFA-producing bacteria) (85, 86).

**FIGURE 1:**
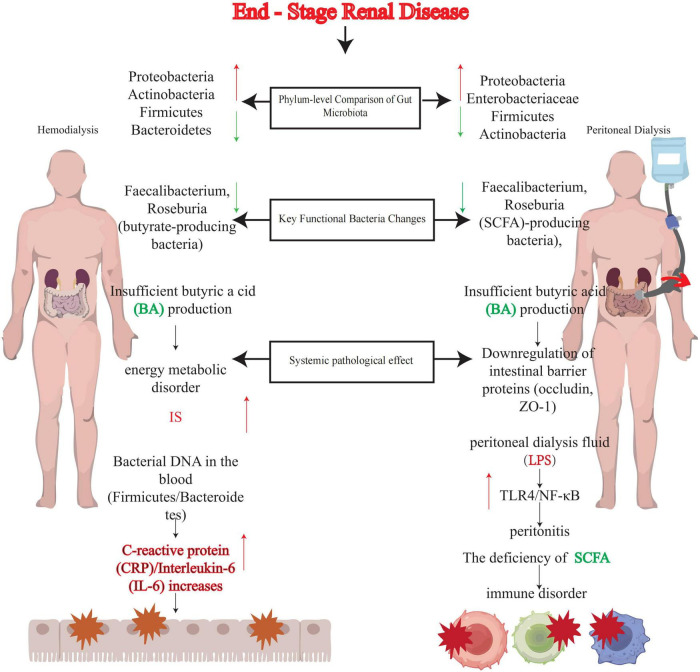
Comparison of the effects of hemodialysis (HD) vs. peritoneal dialysis (PD) on gut microbiota. This figure illustrates the comparison of gut microbiota dysbiosis in end-stage renal disease (ESRD) patients undergoing hemodialysis (HD) or peritoneal dialysis (PD), highlighting phylum-level compositional changes, alterations in functional bacteria, and systemic pathological consequences. Green arrows: Indicate positive regulation or increase. Red arrows: Indicate negative regulation or decrease. SCFA, short-chain fatty acids; BA, butyric acid; IS, indoxyl sulfate; CRP, C-reactive protein; IL-6, interleukin-6; LPS, lipopolysaccharide; TLR4, toll-like receptor 4; NF-κB, nuclear factor κB; ZO-1, zonula occludens-1.

In summary, reductions in SCFA-producing bacteria in both HD and PD patients trigger similar downstream effects (intestinal barrier disruption, bacterial translocation, inflammation), but the specific mechanisms and related complications differ due to variations in treatment modalities (Figure 1). These findings suggest that interventions targeting SCFA metabolism may benefit both patient groups, though strategies must be adjusted according to treatment type. Currently, clinically validated therapeutic approaches include probiotics, prebiotics, synbiotics, plant-based diets, and fecal microbiota transplantation (FMT). By restoring microbial ecology and SCFA homeostasis, these methods hold promise as core components of personalized kidney replacement therapy (KRT) management, synergizing with traditional dialysis regimens.

## 7 Clinical significance and therapeutic approaches

### 7.1 Effects of probiotics, prebiotics, and synbiotics on patients with chronic kidney disease (CKD)

According to definitions established by the International Scientific Association for Probiotics and Prebiotics (ISAPP) consensus and academic literature, probiotics are “live microorganisms that, when administered in adequate amounts, confer a health benefit on the host.” Examples include *Bifidobacterium longum*, *Lactobacillus acidophilus*, and *Enterococcus faecalis*. Prebiotics are “a substrate that is selectively utilized by host microorganisms conferring a health benefit,” such as pectic oligosaccharides, oligosaccharides, inulin, and resistant starch (90). Synbiotics are defined as “a mixture comprising live microorganisms and substrate(s) selectively utilized by host microorganisms that confers a health benefit on the host.” Foods like yogurt, fermented milk, and kefir represent early applications introducing the concept of probiotics and their health benefits into the diets of both healthy individuals and CKD patients (91).

As research into gut microbiota functions deepens, the role of prebiotics in disease is receiving increasing attention. In animal models of CKD, prebiotics (such as resistant starch HAM-RS2 and arabic gum) enhance gut barrier function through fermentation and production of short-chain fatty acids (SCFAs). For instance, Kieffer et al. observed in an adenine-induced CKD rat model that feeding HAM-RS2 reduced cecal pH, altered microbial diversity (e.g., increased Bacteroidetes/Firmicutes ratio), and significantly lowered uremic retention solutes, including serum indoxyl sulfate (IS) (−36%), urinary IS (−66%), and urinary p-cresol (−47%) (92). Vaziri et al. confirmed that fermentable dietary fiber HAM-RS2 attenuated histological abnormalities in CKD rats, including tubular damage, interstitial fibrosis, and inflammatory cell infiltration (93). In human studies, arabic gum supplementation (10–40 g/day) significantly reduced inflammatory markers (e.g., TNF-α, IL-1β) in CKD patients, improved systemic inflammation, and potentially reduced morbidity and mortality (94). Notably, prebiotics may require > 5 g/day to modulate microbial diversity and 15–20 g/day to significantly reduce uremic toxins (95).

Advances in probiotic research reveal that *Lactobacillus johnsonii* may reduce serum creatinine levels and renal fibrosis area in CKD rats—potentially reversing CKD progression—by elevating serum indole-3-carboxaldehyde (IAld) and inhibiting aryl hydrocarbon receptor (AhR) pathway nuclear translocation (96). The same team found that *Lactobacillus* spp. ameliorate membranous nephropathy by suppressing the AhR pathway via tryptophan-derived indole metabolites (97).

Synbiotics demonstrate favorable microbiota-modulating effects in CKD patients. Rossi et al. showed that supplementing 37 Stage IV CKD patients for 6 weeks with a synbiotic (9 probiotic strains + 15 g prebiotic) reduced indoxyl sulfate (IS) and p-cresyl sulfate (PCS) levels by 22%–28%, increased fecal *Bifidobacterium* and *Lachnospiraceae abundance*, decreased *Clostridia*, and improved nutritional status (elevated serum albumin) (98). Another trial found that Stage III–V CKD patients treated for 6 months with a low-protein diet combined with a synbiotic (*Streptococcus thermophilus*, *Lactobacillus acidophilus*, *Bifidobacterium longum*) showed no biochemical improvements but experienced attenuated decline in estimated glomerular filtration rate (eGFR) (99).

McFarlane et al. conducted a 12 months feasibility double-blind placebo-controlled randomized trial in Stage 3–4 CKD adults, demonstrating that long-term synbiotic supplementation is feasible and acceptable. Synbiotics altered fecal microbiota, enriching *Bifidobacterium* and *Blautia* (100). Cosola et al. tested an innovative synbiotic (NATUREN G^®^) in a randomized single-blind placebo-controlled pilot trial involving Stage IIIb–IV CKD patients and healthy controls. Only CKD patients exhibited reduced free IS levels after a 2 months intervention. Though divergent trends in other gut-derived uremic toxins were observed (increasing in placebo, decreasing in synbiotic), changes lacked statistical significance, suggesting CKD-specific effects requiring further validation (101).

However, inconsistencies exist: a study of 30 non-dialysis CKD patients observed no changes in renal function or uremic status after 30 days of probiotics (102). Another 6 weeks synbiotic trial reduced serum p-cresyl sulfate but not serum IS in pre-dialysis CKD patients (103). These discrepancies may relate to strain specificity and individual factors.

In summary, current evidence is insufficient to confirm the efficacy of probiotics, prebiotics, or synbiotics on hard clinical endpoints (e.g., renal function decline rate, cardiovascular events) in CKD patients or establish relative advantages among supplement types. Future rigorously designed randomized controlled trials must define optimal protocols (strain selection, dose optimization, treatment duration) to develop cost-effective gut microbiota-targeted strategies for improving CKD prognosis.

### 7.2 Application of prebiotics, probiotics, and synbiotics in esrd patients

Current evidence indicates that both peritoneal dialysis (PD) and hemodialysis (HD) patients frequently develop gut dysbiosis due to declining residual renal function, dialysis procedures, and constipation (104).

Interventions with prebiotics, probiotics, and synbiotics may alleviate symptoms by modulating the intestinal microenvironment (105).

Research on prebiotics has primarily focused on their potential to clear uremic toxins, but results have shown significant heterogeneity. This heterogeneity may be closely associated with factors such as baseline microbiota characteristics in different populations (e.g., HD vs. PD patients), prebiotic dosage, strain types, and intervention duration.

In HD patients, the effects of inulin exhibit pronounced population specificity and individual variability: Meijers et al. reported that HD patients supplemented with inulin for 4 weeks showed a 20% reduction in serum PCS levels, but no change in IS (106); however, in the trial by Biruete et al. involving 12 HD patients who received 4-week inulin supplementation, no significant changes were observed in fecal or plasma toxin levels, nor were there major alterations in the fecal microbiota (107). In PD patients, the intervention effects of inulin-type prebiotics are directly related to microbiota modulation mechanisms, and intervention duration significantly impacts outcomes. A randomized double-blind crossover trial (*n* = 16) by a Chinese research team showed that continuous ambulatory peritoneal dialysis (CAPD) patients supplemented with inulin-type prebiotics for 24 weeks exhibited a significant decrease in Bacteroidetes abundance, an increase in Firmicutes abundance, and an elevated Firmicutes/Bacteroidetes (F/B) ratio (positively correlated with fecal uric acid degradation capacity). The authors suggested that long-term (24 weeks) intervention may promote uric acid metabolism by enhancing Firmicutes function, thereby reducing serum uric acid levels (108). Another PD patient study (*n* = 21) further found that 36 weeks intervention with inulin-type fructans could target and inhibit the abundance of indole-producing bacteria—*Bacteroides thetaiotaomicron*. This change correlated with reduced fecal indole levels, potentially suppressing the production of uremic toxins (e.g., indoxyl sulfate) (109). However, short-term (3 months) inulin-type fructan intervention (*n* = 22) increased the F/B ratio in PD patients but did not significantly reduce plasma TMAO levels, indicating that intervention duration may be a key factor influencing the toxin-clearing efficacy of inulin-type prebiotics in PD patients (110).

The toxin-clearing effect of resistant starch is more definitive: A 2,010 trial involving 56 HD patients showed that resistant starch supplementation significantly reduced serum indoxyl sulfate (IS) and p-cresyl sulfate (PCS) levels (111); Esgalhado et al. further validated the reduction of IS through resistant starch-enriched biscuit intervention (112). Animal experiments also support its role: CKD rats fed high-amylose resistant starch (HAM-RS2) for 3 weeks exhibited significant improvements in renal interstitial fibrosis, NF-κB activation, and oxidative stress markers, suggesting delayed CKD progression (93).

Probiotics have demonstrated multi-dimensional metabolic benefits. Oral administration of *Bifidobacterium longum* (Bifina strain) can reduce serum homocysteine, triglycerides, and IS levels in HD patients (113); Climent et al. further found that probiotic supplementation may improve depressive symptoms in malnourished HD patients (114). These benefits are likely closely related to probiotics’ modulation of gut microbiota:

In HD patients, probiotic intervention exerts effects by altering microbial composition and the abundance of specific taxa. A Korean randomized double-blind study (*n* = 22) showed that 3 months supplementation with *Bifidobacterium bifidum* BGN4 and *Bifidobacterium longum* BORI significantly increased the relative abundance of *Prevotella*, *Enterococcus*, *Allistipes*, *Clostridium*, *Escherichia-Shigella*, *Klebsiella*, and *Bifidobacterium*, while reducing the abundance of *Bacteroides*, *Faecalibacterium*, *Eubacterium siraeum*, *Tyzzerella*, *Sutterella*, and *Akkermansia* (115). A Chinese research team (*n* = 50) further found that long-term (6 months) supplementation with *Bifidobacterium longum* NQ1501, *Lactobacillus acidophilus* YIT2004, and *Enterococcus faecalis* YIT0072 increased the abundance of Bacteroidaceae and Enterococcaceae, while reducing the abundance of Ruminococ- caceae, Halomonadaceae, Peptostreptococ- caceae, Clostridiales XIII, and Erysipelotrichaceae (largely associated with uremic toxin production) (116). However, Borges’ research team noted that short-term (3 months) probiotic supplementation (e.g., *Streptococcus thermophilus*, *Lactobacillus acidophilus*, and *Bifidobacterium longum*) had limited effects on microbiota structure in HD patients, with no significant differences in microbiota band counts (reflecting composition) between the probiotic and placebo groups (117).

In PD patients, probiotic intervention primarily modulates phylum-level microbiota and reduces opportunistic pathogens. A single-center study by Liu et al. (*n* = 57) showed that 12 weeks supplementation with *Lactobacillus paracasei* N1115 (containing fructooligosaccharides) significantly increased Firmicutes abundance, decreased Bacteroidetes abundance, and reduced the abundance of opportunistic pathogens (e.g., *Fusobacterium*, *Bilophila*). Although microbiota diversity (α/β) showed no significant changes, these alterations correlated with improvements in gastrointestinal symptoms (e.g., dyspepsia, constipation) (118).

Despite the metabolic and symptomatic benefits of probiotics through microbiota modulation, their role in cardiovascular disease (CVD) prevention remains controversial: Some studies suggest they may reduce CVD risk via cholesterol regulation, blood pressure modulation, and anti-inflammatory effects (119, 120), but mechanisms such as strain-specific immune regulatory pathways remain unclear, and data on hard endpoints (e.g., mortality, myocardial infarction) are lacking.

Synbiotic formulations (probiotic-prebiotic combinations) show unique advantages. A 2 weeks regimen combining *Lactobacillus casei* Shirota, *Bifidobacterium breve* Yakult, and galactooligosaccharides significantly reduced serum PCS in HD patients (121). Iranian researchers observed superior reductions in IL-6, CRP, and endotoxemia with synbiotics versus probiotics alone (122, 123). Paradoxically, another Iranian study reported synbiotic-induced IS elevation in HD patients (122).

The peritoneal microenvironment in PD patients may reshape intervention efficacy. Continuous dialysate exposure alters intestinal osmolarity and microbial colonization, though evidence remains scarce. Preliminary data suggest probiotics (*B. longum*, *Lactobacillus plantarum*) in PD patients reduce serum endotoxin, proinflammatory cytokines (TNF-α, IL-6, IL-5), and elevate anti-inflammatory IL-10 while preserving residual renal function (124). Additional studies highlight oral probiotics’ ability to lower serum uric acid (108) and endotoxin (125) in PD patients, affirming the safety and gastrointestinal symptom-relieving benefits of pre/pro/synbiotic supplementation.

In summary, current research is constrained by small sample sizes, short intervention durations, and strain/dosage variability. Although systematic reviews suggest prebiotics modestly reduce serum urea and synbiotics modulate microbial composition, the lack of hard clinical endpoint data—particularly in PD cohorts—remains the foremost translational challenge (Table 1).

**TABLE 1:** Summary of clinical trials on the use of prebiotics, probiotics, and synbiotics supplements in d-stage renal disease (ESRD) patients.

Study population	References	Country/ region	Type of study	Sample size	Detailed characteristics of the sample	Probiotic/ prebiotic/ synbiotic type and duration of intervention	Details of the intervention	Observation indicators	Screening period	Adverse reaction	Remarks	Microbiota analysis results
Hemodialysis patients	(126)	United States	Randomized, double-blind, placebo-controlled cross-over trial	28	Aged between 18 and 80, diagnosed with chronic kidney disease stage V (end-stage renal disease) and currently receiving hemodialysis treatment.	30 billion colony-forming units (CFU) of *Streptococcus thermophilus* KB 19, *Lactobacillus acidophilu* KB 27, and *Bifidobacterium longum* KB 31 duration: 4 months	The patient takes two capsules three times a day.	Measurable improvements in quality of life (based on the modified SF36 questionnaire), levels of biochemical markers (such as urea and creatinine), hematological values (CBC), and liver function levels.	April 2011	There was one serio us adverse event with a fatal outcome, which was unrelated to the study protocol.	Renadyl (a strain-specific probiotic preparation) appears to be safely used in hemodialysis patients with ESRD (end-stage renal disease).	–
Hemodialysis patients	(127)	Mexico	Double-blind, placebo-controlled, randomized clinical trial	42	Aged 18 years and above, who have received hemodialysis three times a week for at least 3 months prior to the start of the study.	*Lactobacillus acidophilus* NCFM and *Bifidobacterium lactis* Bi-07 duration: 2 months	Twenty-two patients were randomly assigned to the intervention group (nutritional counseling + symbiotic gel), and 20 patients were randomly assigned to the control group (nutritional counseling + placebo).	The presence and monthly episodes of GIS were evaluated, along with biochemical parameters, inflammatory markers, and nutritional status.	–	Three patients voluntarily withdrew due to diarrhea, including 2 in the control group and 1 in the intervention group.	The administration of symbiotic gel is a safe and simple method to improve common GIS (gastrointestinal symptoms) in dialysis patients.	–
Hemodialysis patients	(123)	Iran	Double-blind, randomized, placebo-controlled clinical Trial	75	Aged between 30 and 65 years, dialyzed via arteriovenous fistula, and received hemodialysis treatment three times a week for at least 3 months prior to the start of the study.	The synbiotic powder contain *Lactobacillus acidophilus* T16 (IBRC-M10785), *Bifidobacterium bifidum* BIA 6, *Bifidobacterium lactis* BIA-6, and *Bifidobacterium longum* LAF-5, and also includes 15 g of prebiotics consisting of three types of fibers: 5 g of fructooligosaccharides (FOS), 5 g of galactooligosaccharides (GOS), and 5 g of inulin, with a duration of 12 weeks.	Patients were randomly assigned to one of three groups. The first group received synbiotics (*n* = 25), the second group received probiotics (*n* = 25), and the third group received a placebo (*n* = 25).	Changes in serum inflammatory markers, endotoxin, and anti-HSP70 after probiotic and synbiotic supplement interventions.	–	–	The administration of synbiotics was more effective than probiotics in improving inflammatory markers, endotoxin, and anti-HSP70 serum levels.	–
Hemodialysis patients	(128)	Iran	Parallel clinical trial	36	Aged 17 years or older, receiving dialysis for 4 h, three times a week; positive C-reactive protein (CRP) result; no severe hyperparathyroidism; no active bleeding or surgery in the past 3 months; no hemoglobin (Hb) disorders.	Contains *Lactobacillus acidophilus*, *Bifidobacterium*, and *Streptococcus thermophilus* duration: 12 weeks	Patients were randomly divided into two groups. The intervention group (*n* = 18) took 500 mg of probiotic supplement daily, while the control group (*n* = 18) received a placebo.	Hemoglobin (Hb) concentration (anemia index) and serum C-reactive protein (CRP) levels in the probiotic group and placebo group before and after intervention.	23 August 2014, ending 22 November 2014.	–	Probiotic supplementation reduced Hb fluctuations in hemodialysis patients but did not result in a significant increase in Hb levels.	–
Hemodialysis for diabetic patients	(129)	Iran	Parallel Randomized Double-Blind Placebo-Controlled Clinical Trial	60	–	Probiotics: *Lactobacillus acidophilus*, *Lactobacillus casei*, and *Bifidobacterium bifidum* duration: 12 weeks	Patients were randomly assigned to receive either capsules containing probiotics patients were randomly assigned to receive either capsules containing probiotics (*Lactobacillus acidophilus*, *Lactobacillus casei*, and *Bifidobacterium bifidum*) or a placebo.	Insulin metabolism parameters, blood lipid indices, inflammatory biomarkers, oxidative stress indices	29 February 2016 to 5 March 2016.	–	Probiotic supplementation for 12 weeks in diabetic hemodialysis patients has beneficial effects on glucose homeostasis parameters and some biomarkers of inflammation and oxidative stress.	–
Hemodialysis patients	(130)	Iran	Randomized controlled double-blind clinical trial	42	Aged over 20 years, having received chronic maintenance hemodialysis for at least 3 months, with diverse medical histories including diabetes or hypertensive nephropathy, nephrotic syndrome, and glomerulonephritis.	*Lactobacillus rhamnosus*, duration: 4 weeks	The intervention group took one capsule containing *Lactobacillus rhamnosus* after meals daily, while the control group took one placebo capsule made from infant formula after meals daily.	The effect of supplements on serum uremic toxin (p-cresol and phenol) levels.	–	–	Probiotics (*Lactobacillus rhamnosus*) can effectively reduce uremic toxins (p-cresol and phenol) in hemodialysis patients.	–
Hemodialysis patients	(117)	Brazil	Randomized, double-blind, placebo-controlled study	46	Inclusion of patients aged 18 years and older who have received hemodialysis (HD) for at least 6 months.	*Streptococcus thermophilus, Lactobacillus acidophilus*, and *Bifidobacterium longum*, duration: 3 months.	Patients were randomly divided into two groups: the probiotic group (*n* = 23; containing *Streptococcus thermophilus*, *Lactobacillus acidophilus*, and *Bifidobacterium longum*, 90 billion colony-forming units daily) and the placebo group (*n* = 23).	Inflammatory markers (C-reactive protein and interleukin-6), plasma uremic toxin levels (indoxyl sulfate, p-cresyl sulfate, and indole-3-acetic acid), fecal pH, and gut microbiota characteristics.	–	–	Probiotic supplementation failed to reduce the levels of uremic toxins and inflammatory markers.	No significant differences in intestinal microbiota band numbers (reflecting microbiota composition) were observed between the probiotic group and placebo group at both baseline and post-intervention, suggesting that short-term probiotic supplementation may not significantly alter the microbiota structure in patients.
Hemodialysis patients	(115)	South Korea	Randomized, double-blind, placebo-controlled study	22	Patients aged ≥ 18 years who have undergone maintenance dialysis treatment for more than 3 months.	*Bifidobacterium bifidum* BGN4 and *Bifidobacterium longum* BORI. duration: 3 months.	Patients are required to take two sachets daily for three consecutive months (each sachet is a 2 g probiotic mixture containing 7.0 × 109 colony-forming units (CFU) of *Bifidobacterium bifidum* BGN4 and 2.0 × 109 CFU of *Bifidobacterium longum* BORI per gram).	Changes in the microbiome and fecal short-chain fatty acids (SCFA), various inflammatory parameters, serum calprotectin levels, and cytokine responses to lipopolysaccharide (LPS) stimulation before and after probiotic supplementation.	For the period November–December 2018.	One patient was diagnosed with calculous cholecystitis, underwent laparoscopic cholecy- stectomy, and received systemic antibiotic treatment. The second patient developed community-acquired pneumonia one month after starting probiotic supplemen- tation and was discharged after 5 days of systemic antibiotic therapy.	Probiotic supplementation can alleviate systemic inflammatory responses in hemodialysis patients, and this effect is associated with an increase in regulatory T cells and a decrease in pro-inflammatory monocytes.	After probiotic supplementation, the relative abundances of Prevotella, *Enterococcus*, Alistipes, Clostridia, Escherichia-Shigella, Klebsiella, and *Bifidobacterium* increased, while those of *Bacteroides*, Faecalibacterium, Eubacterium siraeum, Tyzzerella, Sutterella, and Akkermansia decreased.
Hemodialysis patients	(118)	China	Single-center, double-blind, randomized, placebo study	50	Aged 18–70 years, diagnosed with CKD stage V (currently receiving hemodialysis treatment for more than 3 months).	The probiotic powder contains: *Bifidobacterium longum* NQ1501, *Lactobacillus acidophilus* YIT2004, and *Enterococcus faecalis* YIT0072, with a duration of 6 months.	Randomly assigned to the probiotic group or placebo group at a 1:1 ratio.	The response of gut microbiome, serum and fecal metabolome, serum albumin and endotoxin, endothelial activation markers and inflammatory markers to intervention.	9 December 2016 to 31 May 2017	–	Probiotics do offer benefits in improving intestinal imbalance and reducing exposure to multiple uremic toxins in HD (hemodialysis) patients.	Probiotics increased the abundances of Bacteroidaceae and Enterococcaceae, while decreasing the abundances of Ruminococcaceae, Halomonadaceae, Peptostreptococ- caceae, Clostridiales Family XIII. Incertae Sedis, and Erysipelotri- chaceae.
Hemodialysis patients	(122)	Iran	Randomized, double-blind, placebo-controlled study	48	Regardless of age, gender, and race, receive HD treatment three times a week, with each session lasting no less than 4 h.	Contains probiotics (*Lactobacillus casei*, *Lactobacillus acidophilus*, *Lactobacillus rhamnosus*, *Lactobacillus bulgaricus*, *Bifidobacterium breve*, *Bifidobacterium longum*, *Streptococcus thermophilus*) and prebiotic fructooligosaccharides (FOS), for a period of 8 weeks.	Randomly assign two groups, synbiotic group: *n* = 21; placebo group: *n* = 21.	Serum urea, creatinine, liver enzymes, high-sensitivity C-reactive protein, sodium, potassium, phosphorus, blood pressure, albumin levels, indoxyl sulfate, and parathyroid hormone levels	From October 2015 to December 2016	–	After 2 months of combined prebiotic and probiotic treatment, the circulating levels of indoxyl sulfate and PTH in the intervention group increased significantly.	–
Hemodialysis patients	(109)	Brazil	Randomized single-blind and placebo-controlled intervention study.	58	Patients over 18 years of age receiving standard hemodialysis treatment	The synbiotic group - 100 ml probiotic dairy beverage + 40 g extruded sorghum flakes, or the control group - 100 ml pasteurized milk + 40 g extruded corn flakes, for 7 weeks.	Randomly assigned to the synbiotic group: *n* = 29; or the control group: i = 29.	The difference in IS concentration was used as the primary outcome.	–	–	The synbiotic meal reduced serum uremic toxins in HD subjects after 7 weeks of intervention.	–
Hemodialysis patients	(110)	India	Double-blind randomized controlled clinical trial (RCT)	60	Patients aged 18 and above receiving standard hemodialysis treatment, twice a week, 5 h each time, for at least 3 months, and patients with gastrointestinal discomfort (i.e., defecation difficulty, hard stool texture, or defecation frequency less than three times per week	Synbiotic group: 60 synbiotic capsules [each containing *Lactobacillus acidophilic* and *Bifidobacterium longum*, as well as fructooligosaccharides (FOS)]; placebo group: 60 placebo capsules (each containing lactose). The daily dosage is 2 capsules, to be taken before breakfast in the morning. The intervention period is 2 months.	Randomly assigned to the synbiotic group: *n* = 30; or the control group: *n* = 30.	The effects on indoxyl sulfate levels, constipation symptoms, and constipation-related quality of life	August-December 2020	–	2 months synbiotic supplementation did not reduce indoxyl sulfate toxin levels, but improved constipation in patients receiving chronic hemodialysis.	–
Hemodialysis patients	(114)	Spain	A randomized multicenter parallel clinical study	31	Malnourished adult subjects (> 18 years old) who had received unchanged hemodialysis treatment within 3 months before enrollment and during the 6 months intervention period.	Probiotics vs. Placebo: probiotic capsules (380 g per capsule) with live bacteria: *Bifidobacterium breve* CNCM I-4035; *Bifidobacterium animalis subsp. Lactis* CECT 8145; *Lactobacillus paracasei* CNCM I-4034. Placebo: capsules visually identical to probiotic capsules, specific ingredients not mentioned. ONS product (renacare): specifically designed for malnourished hemodialysis patients, featuring high energy (2 kcal/mL), high protein, and rich in functional nutrients. Intervention duration: 6 months.	11 subjects in control group; 10 subjects in ONS + placebo group; 10 subjects in ONS + probiotics group.	Blood biomarkers, psychological questionnaires.	–	–	Probiotics combined with nutritional supplements can reduce intestinal permeability biomarkers in malnourished hemodialysis patients, and improve depressive symptoms and quality of life.	–
Hemodialysis patients	(131)	China-Taiwan	A randomized double-blind placebo-controlled clinical trial (RCT)	56	Adult subjects receiving hemodialysis	The probiotic group patients took a mixture containing three high-dose freeze-dried live bacterial strains twice daily: *Lactococcus lactis subsp lactis* LL358, *Lactobacillus salivarius* LS159, and *Lactobacillus pentosu* LPE588, for 6 months.	Randomly assigned to the probiotic group: *n* = 28; or the control group: *n* = 28.	Hemoglobin levels, blood urea nitrogen, blood glucose, serum p-cresyl sulfate, inflammation and microbial translocation markers	–	–	Supplementation with probiotics had no significant effect on the cholesterol-to-triglyceride ratio.	–
Hemodialysis-dependent diabetic patients	(132)	Iran	A randomized, double-blind, placebo-controlled clinical trial	60	Diabetic patients aged 18 to 80 years on hemodialysis	Synbiotics: containing *Lactobacillus acidophilus*, *Lactobacillus casei*, *Bifidobacterium*, and 0.8 g/day inulin. Placebo: corn starch, Duration: 12 weeks	Synbiotic group: *n* = 30, control group: *n* = 30	Insulin metabolic parameters, lipid profile indicators, inflammatory biomarkers, oxidative stress markers	November 2017 to February 2018	–	Synbiotic supplementation has beneficial effects on glycemic control, inflammatory markers, and oxidative stress indices in diabetic hemodialysis (HD) patients.	–
Hemodialysis patients	(133)	Brazil	A randomized double-blind trial	21	Patients over 18 years old receiving hemodialysis	Probiotic group: containing *Streptococcus thermophilu* (KB19), *Lactobacillus acidophilus* (KB27), and *Bifidobacterium longum* (KB31), placebo: composed of wheat germ, duration: 3 months, dosage: three capsules daily for each group	Placebo group: *n* = 10, probiotic group: *n* = 11,	LC-MS/MS detection of plasma TMAO, choline, and betaine levels	–	–	Short-term probiotic supplementation does not affect plasma TMAO levels in HD patients.	–
Continuous ambulatory peritoneal dialysis	(107)	China	Randomized, double-blind, placebo-controlled cross-over trial	16	Aged 18 years or older, receiving continuous ambulatory peritoneal dialysis (CAPD) treatment for more than 3 months, without a history of diabetic nephropathy, and not pregnant	Inulin-type prebiotics (mixture of inulin and fructooligosaccharides), duration: 24 weeks	Thirty-three participants were randomly assigned to two study groups, with the order of placebo (maltodextrin) and prebiotic intervention switched between groups. The intervention was 10 g per day, dissolved in warm water and taken orally.	Serum uric acid (UA) levels, fecal UA degradation capacity, fecal microbial composition and function.	For the period from 2017 to 2020	–	Inulin-type prebiotics are promising therapeutic candidates for reducing serum uric acid (UA) levels in patients with renal failure, and this uric acid-lowering effect may be attributed to the gut microbial degradation of UA.	Phylum level: Bacteroidetes: decreased abundance. Firmicutes: increased abundance.
Continuous ambulatory peritoneal dialysis	(116)	China	Single-Center, prospective, randomized, double-blind, placebo-controlled study	57	Receiving stable continuous ambulatory peritoneal dialysis (CAPD) treatment for more than 3 months, Aged over 18 years.	Fructooligosaccharides (FOS) (with an addition amount > 80%), maltodextrin, and *Lactobacillus paracasei* N1115. For a duration of 12 weeks.	Patients were assigned to the probiotic (PR) group and placebo (PL) group at a 2:1 ratio. The probiotic group received probiotics, while the placebo group received placebo.	Changes in the diversity, abundance, and composition of the gut microbiota, as well as fecal short-chain fatty acid (SCFA) levels in patients after intervention. Serological indicators and Gastrointestinal Symptom Rating Scale (GSRS) scores.	May 2020 to December 2022	–	A 12 weeks probiotic supplementation had no significant effect on the primary outcomes of short-chain fatty acids (SCFA) and bacterial diversity, but altered the composition of the gut microbiota in peritoneal dialysis (PD) patients.	Phylum level: Firmicutes showed a significant increase in abundance; Bacteroidetes showed a significant decrease in abundance. Specific genera: Abundances of opportunistic pathogens (e.g., Fusobacterium, Bilophila) significantly decreased. Diversity: no significant differences in intestinal microbial diversity (α/β diversity) were observed between the two groups.
Peritoneal dialysis patients	(134)	China	Randomized controlled and open-label trial	116	Patients aged between 18 and 75 years who are receiving peritoneal dialysis (PD) in outpatient settings and have undergone PD treatment for more than 3 months.	The probiotic capsules are composed of *Bifidobacterium longum*, *Lactobacillus bulgaricus*, and *Streptococcus thermophilus*. Duration: 2 months	Patients were randomly assigned to one of two parallel groups at a 1:1 ratio.	High-sensitivity C-reactive protein and interleukin-6 levels, serum albumin levels, upper arm circumference, and triceps skinfold thickness before and after the intervention	For the period March 2017 to February 2018	–	Probiotics can significantly reduce the levels of serum high-sensitivity C-reactive protein (hs-CRP) and interleukin-6 (IL-6) in peritoneal dialysis (PD) patients, while increasing serum albumin levels, upper arm circumference, and triceps skinfold thickness.	–
Peritoneal dialysis (PD) patients	(124)	China	A randomized, double-blind, placebo-controlled trial	39	Patients aged > 18 years undergoing PD	Probiotic capsules containing *Bifidobacterium* sp. A218, *Bifidobacterium catenulatum* A302, *Bifidobacterium longum* A101, and *Lactobacillus plantarum* A87; placebo group received maltodextrin capsules for 6 months.	The intervention group received 1 probiotic capsule daily (*n* = 21), while the placebo group received 1 placebo capsule daily (*n* = 18).	Inflammatory markers and serum endotoxin levels measured pre- and post-intervention.	July 2011–June 2012	–	Probiotics significantly reduced serum endotoxin, pro-inflammatory cytokines (TNF-α, IL-6, IL-5), and increased anti-inflammatory cytokine (IL-10) levels in PD patients. Residual renal function was also preserved.	–
Continuous ambulatory peritoneal dialysis (CAPD) patients	(135)	China	A randomized, double-blind, placebo-controlled crossover trial	21	Undergoing CAPD for > 3 months, Aged 18–65 years able to provide informed consent.	Placebo: Maltodextrin (Roquette), identical in appearance, packaging, and administration to prebiotics. Prebiotics: Inulin-type fructan (50:50 mix of long-chain inulin and fructooligosaccharides, Synergy1, Orafti). Duration: 36 weeks.	21 stable patients randomized to two intervention sequences: prebiotics first → placebo later. Placebo first → prebiotics later.	Intestinal microbiome. Fecal indole and p-cresol level. Abundance of indole-producing and p-cresol-producing bacteria. Serum indoxyl sulfate (IS) and p-cresyl sulfate (pCS) level.	–	–	Inulin-type fructan intervention inhibited elevation of intestinal microbial indole levels and reduced intestinal pH.	Inulin-type oligosaccharide intervention showed no significant statistical impact on gut microbiota composition at phylum and genus levels, but potentially affected intestinal metabolism by quantitatively reducing the abundance of *Bacteroides thetaiotaomicron* (indole-producing bacteria).
Continuous ambulatory peritoneal dialysis (CAPD) patients	(136)	China	A randomized, double-blind, placebo-controlled crossover trial	22	Undergoing CAPD for> 3 months. Aged 18–65 years.	Prebiotics: Inulin-type fructan (10 g daily for 3 months). Placebo: (Not specified, but implied as matching prebiotic in appearance/dosage per crossover design]	22 stable patients randomized to two intervention sequences: Prebiotics first → placebo later. Placebo first → prebiotics later.	Fecal and plasma trimethylamine (TMA). Plasma trimethylamine N-oxide (TMAO). Daily urinary excretion of TMAO. Dialytic clearance rate of TMAO.	–	–	Daily 10 g inulin-type fructan intervention for 3 months was insufficient to reduce plasma TMAO levels in CAPD patients, but improved gut microbiota composition.	Inulin-type fructan intervention increased the Firmicutes/ Bacteroidetes (F/B) ratio in the gut microbiome.
Automated peritoneal dialysis (APD) patients	(137)	Brazil	A randomized, double-blind, placebo-controlled crossover trial	43	Patients aged 18–80 years, with ≥ 3 months of dialysis duration and strict adherence to dialysis protocols.	Placebo: waxy corn starch. Prebiotic: unripe banana flour (UBF) containing ∼48% resistant starch, 7% other fibers, and trace plant sterols/polyphenols. Duration: 12 weeks (4 weeks treatment per group +4 week washout period)	Randomized to sequential UBF (21 g/day) and placebo (waxy corn starch, 12 g/day) groups with 4 weeks washout between phases.	Serum total indoxyl sulfate (IS), p-cresyl sulfate (pCS), indole-3-acetic acid (IAA), and their free form levels.	May 2018–September 2018	–	UBF did not significantly affect serum IS, pCS, and IAA levels in PD patients. A reduction in IS was observed only in the subgroup with strict adherence to 21 g/day dosage.	–

### 7.3 Translational potential and clinical therapeutic approaches of plant-based diets in peritoneal dialysis (PD) and hemodialysis (HD) patients

Plant-based diets (PBDs), characterized by a focus on unprocessed or minimally processed plant foods such as whole grains, legumes, nuts, fruits, and vegetables, while strictly limiting animal products (meat, fish, eggs, dairy) and processed foods (e.g., refined vegetable oils, plant-based meat substitutes), offer multifaceted benefits in dialysis populations. These diets are distinguished by two core components: dietary fibers and phytochemicals (138).

Dietary fibers in plant-based foods include insoluble fibers (hemicellulose, cellulose, lignin) and soluble carbohydrates (pectins, gums, mucilages), as well as indigestible resistant starch and oligosaccharides (139). These non-digestible components possess strong water-absorbing properties, expanding fecal volume within the colon to mechanically stimulate intestinal peristalsis and accelerate bowel transit time (140). Additionally, anaerobic microbial fermentation of these fibers generates gaseous byproducts and short-chain fatty acids (SCFAs)—primarily acetate, propionate, and butyrate—which play critical roles in maintaining human health and regulating gut homeostasis (141).

Phytochemicals, bioactive non-nutrient compounds found in plants, exhibit anti-inflammatory and antioxidant properties. A Swedish cross-sectional study of dialysis patients revealed that higher proportions of linoleic acid (a plant-derived fatty acid) in plasma were inversely correlated with IL-6 levels and all-cause mortality, suggesting clinical benefits from increased plant-based oil consumption (142). Polyphenols, another class of phytochemicals widely distributed in plants, mitigate cardiovascular risk—a leading cause of death in dialysis patients—by enhancing nitric oxide (NO)-mediated endothelial function and reducing low-density lipoprotein (LDL) oxidation, thereby preventing atherosclerosis. In cardiomyocytes, polyphenols suppress the expression and production of inflammatory markers, exerting anti-inflammatory effects (143).

The alkaline nature of plant-based foods, particularly fruits and vegetables, reduces dietary acid load. Animal-derived foods (e.g., cheese, meat, fish) typically generate higher acid loads compared to plant-based alternatives (144). PBDs also reshape the spectrum of uremic toxins through microbial modulation. Stanford et al. further observed that HD patients adhering to PBDs exhibited reduced abundances of IS/PCS-associated pathogenic bacteria (*Haemophilus, Haemophilus parainfluenzae*), while increased animal fat and sweets consumption correlated with elevated toxin levels (145).

Clinical studies validate survival benefits associated with PBDs. A retrospective analysis of 884 PD patients revealed that higher plant protein intake (> 57.5% vs. < 47.7% of total protein) was linked to a 24% reduction in mortality. Each 10% increase in plant protein intake correlated with 71% and 89% lower risks of all-cause and cardiovascular mortality, respectively (146). Similarly, a multinational cohort of 8,078 HD patients demonstrated that the highest tertile of fruit/vegetable intake was associated with 20% lower all-cause mortality (95% CI: 9%–29%) and 23% reduced non-cardiovascular mortality (95% CI: 9%–34%) compared to the lowest tertile (147).

High fiber intake improves clinical outcomes through dual metabolic and anti-inflammatory pathways. A cross-sectional study of 52 PD patients found that daily fiber intake > 12.2 g reduced serum and dialysate IL-6 and CRP levels (148). In a 45 months follow-up of 881 PD patients, higher fiber consumption was associated with increased serum albumin over time. Among non-diabetic patients, each 1 g/day increase in fiber intake correlated with a 13% lower all-cause mortality, though no cardiovascular mortality benefits were observed in the overall cohort or subgroups (149).

A major concern in advocating PBDs for dialysis patients is the perceived risk of hyperkalemia. Despite clinical recommendations to restrict dietary potassium, current evidence does not support an increased hyperkalemia risk with plant-based diets. Data from 8,043 HD patients in Europe and South America showed no significant association between dietary potassium intake and serum potassium levels or all-cause mortality (150). Similarly, a PD cohort study (*n* = 881) found no independent correlation between dietary potassium intake and mortality over 45 months (151) (Table 2). Potassium-rich plant foods (e.g., bananas, spinach) may confer cardiorenal protection through urinary alkalinization and enhanced sodium excretion, though their long-term risk-benefit ratio in dialysis populations requires further validation (152).

**TABLE 2:** Summary of clinical trials on plant-based diets in peritoneal dialysis (PD) and hemodialysis (HD) patients.

Study population	References	Type of study	Research design	Specific components and intervention cycle of dietary intervention	Observation indicators	Adverse reaction	Remarks
22 hemodialysis patients	(145)	Cross-sectional analysis	Two methods were used to assess dietary quality: (1) The overall Plant-based Diet Index (PDI), Healthy Plant-based Diet Index (hPDI), and Unhealthy Plant-based Diet Index (uPDI); (2) Classification of food category intake. | 10 weeks.	Assessing dietary quality. Duration: 10 weeks	Investigate the associations among dietary quality, gut microbiota profiles, and key uremic toxins in adults receiving hemodialysis treatment.	–	For adults undergoing hemodialysis, dietary quality and food choices may influence the production of uremic toxins by the gut microbiota.
8,078 hemodialysis patients	(147)	Prospective cohort study	Study on the dietary intake, mortality and hospitalization of adult patients with end - stage kidney disease (ESKD) receiving hemodialysis treatment through dietary assessment analysis	–	The associations among the fruit and vegetable intake of patients receiving hemodialysis treatment, and all - cause, cardiovascular, and non - cardiovascular mortality.	–	In the hemodialysis population, fruit and vegetable intake is generally low. Higher intake is associated with lower all-cause and non-cardiovascular mortality.
884 peritoneal dialysis patients	(146)	Prospective cohort study	All patients visited a doctor at least every 3 months. All patients were followed up until death, conversion to hemodialysis (HD), kidney transplantation, loss to follow-up, or the end of the study. Laboratory indices and nutritional parameters were measured periodically and repeatedly, and their time-averaged values were calculated.	**–**	The association between the proportion of plant-based protein in total protein intake among patients and all-cause mortality as well as cardiovascular disease mortality.	–	A diet with a higher proportion of plant-based protein to total protein is associated with lower all-cause mortality and CVD mortality in PD patients, and this association is more pronounced in female patients, elderly patients, and those without hypoalbuminemia.
52 peritoneal dialysis patients	(148)	Observational and cross-sectional studies	Included were adult patients who had been clinically stable on peritoneal dialysis (PD) for more than 30 days. All dietary data were obtained through interviews.	Fiber intake was assessed by means of a dietary survey and calculated using the DietPro program 5.6i.	The association between dietary fiber intake and interleukin (IL)-1β, IL-6, tumor necrosis factor-α (TNF-α), monocyte chemoattractant protein-1 (MCP-1), B-cell activating factor (BAFF), and plasminogen activator inhibitor-1 (PAI-1) levels in serum and peritoneal effusion.	–	Peritoneal dialysis (PD) patients often have insufficient dietary fiber intake, which appears to be associated with inflammatory responses, particularly intraperitoneal inflammatory responses.
881 peritoneal dialysis patients	(149)	Single-center prospective cohort study	All patients were followed up until death, conversion to hemodialysis, kidney transplantation, or the end of the study.	–	The association between dietary fiber and mortality risk in dialysis patients.	–	Higher dietary fiber intake appears to have a protective effect against all-cause mortality in non-diabetic peritoneal dialysis (PD) patients.
56 hemodialysis patients	(111)	Single-blind prospective randomized trial	Fifty-six patients were randomly assigned, with 28 allocated to the fiber group and 28 to the control group.	Hi - maize 260, a high - amylose corn starch, contains approximately 40% digestible starch and 60% resistant starch (indigestible fiber), with a 6 weeks intervention period.	Plasma levels of indoxyl sulfate and p-cresyl sulfate before and after intervention.	–	Increasing dietary fiber intake in hemodialysis patients may reduce plasma levels of colon-derived solutes such as indoxyl sulfate and potentially p-cresyl sulfate, without the need for intensified dialysis treatment.
124 hemodialysis patients	(153)	Randomized placebo-controlled trials	Hemodialysis patients were randomly selected and given 10 g/day, 20 g/day of fiber, or a placebo for 6 weeks. Anthropometric indices and 24 h dietary recall intake were assessed.	Water-soluble fiber (fermentable rate > 75%), 6 weeks (randomized controlled trial), plus 1 week for baseline establishment.	Lipid profiles, oxidative status, and systemic inflammatory status before and after intervention.	–	Dietary fermentable fiber supplements improved lipid profiles and oxidative status, and reduced systemic inflammatory status in hemodialysis patients.

### 7.4 Mechanisms and Clinical translation of fecal microbiota transplantation (fmt) in dialysis patients

Fecal microbiota transplantation (FMT), by transplanting gut microbiota from healthy donors, offers targeted intervention for microbial dysbiosis in dialysis patients, aiming to reduce uremic toxin accumulation, systemic inflammation, and metabolic disturbances. Animal studies have revealed differential effects of FMT on toxin clearance. In adenine-induced CKD mouse models, FMT significantly reduced serum p-cresyl sulfate (PCS) levels and improved glucose tolerance, though no changes in renal function were observed (154). In streptozotocin-induced diabetic nephropathy mouse models, FMT lowered lipopolysaccharide (LPS) and trimethylamine N-oxide (TMAO) concentrations while increasing beneficial short-chain fatty acids (SCFAs) such as propionate and butyrate (155). In a traumatic brain injury (TBI) rat model, FMT rescued gut microbiota alterations induced by TBI at 8 days post-injury, alleviated neurological deficits, reduced fecal trimethylamine (TMA) levels, and decreased TMAO concentrations in both the ipsilateral brain and serum (156). Bastos et al. demonstrated that FMT prevented weight gain, reduced proteinuria, and lowered TNF-α levels in BTBR ob/ob mice, confirming its non-pharmacological therapeutic potential for diabetic nephropathy (157) (Table 3).

**TABLE 3:** Experimental and clinical studies of fecal microbiota transplantation (FMT) in chronic kidney disease (CKD) patients.

References	Type of study	Disease models	Research design	Adverse reaction	Remarks
(161)	Case reports	Membranous nephropathy	On day 0 and day 28, endoscopic administration was performed twice. Under anesthesia, 150 mL of fecal suspension was injected into the patient’s small intestine through an endoscope, and 350 mL was injected into the colon.	–	Fecal microbiota transplantation (FMT) is a promising biotherapy for membranous nephropathy
(160)	Case reports	IgA nephropathy	Case 1: a total of 40 fecal microbiota transplantation (FMT) procedures were consecutively performed (200 mL daily, 5 days per week), followed by an additional 57 procedures over the next 5 months (200 mL daily, 10 to 15 days per month). Case 2: sixty treatments were administered via trans-enteral tube (TET) over 6 months (200 mL daily, 10–15 days per month), with a 6 months follow-up.	–	After receiving intensive fresh fecal microbiota transplantation (FMT) for 6–7 months, partial clinical remission was achieved with stable renal function.
(162)	Case reports	Peritoneal dialysis patients	A case of successful fecal microbiota transplantation via colonoscopy. The patient received multiple fecal microbiota transplantations to enhance bacterial colonization.	–	Fecal microbiota transplantation (FMT) may be an important and promising therapeutic strategy for treating Clostridioides difficile infection in patients receiving peritoneal dialysis.
(157)	Animal studies	BTBR ob/ob DKD mouse model	For each sample from BTBR wild-type donors, 300 mg of feces collected from various intestinal segments was dissolved in 500 μL of phosphate-buffered saline. The supernatant of the sample (approximately 300 μL) was injected into the intestine via a rectal route using a polyethylene probe.	–	Fecal microbiota transplantation is a safe therapeutic method that can inhibit weight gain in BTBRob/ob mice, reduce proteinuria, decrease the local expression of tumor necrosis factor-α (TNF-α) in the ileum and ascending colon, and may improve insulin resistance.
(159)	Animal studies	STZ-induced diabetic kidney disease (DKD) rat model	The suspension was thawed, mixed, and slowly injected into the stomach of diabetic rats through a catheter.	–	Fecal microbiota transplantation (FMT)-mediated modulation of gut microbiota can effectively downregulate the activation of the GPR43 signaling pathway and protect podocytes from damage in diabetic rats by reducing serum acetate levels.
(154)	Animal studies	Adenine-induced CKD mouse model	The mice were randomly divided into three groups: control group, chronic kidney disease (CKD) group, and CKD + fecal microbiota transplantation (FMT) group. After 4 weeks, the mice in the CKD group received FMT from healthy mice, while the control group was treated with phosphate-buffered saline (PBS).	–	Fecal microbiota transplantation (FMT) limits the accumulation of uremic toxins produced by the intestinal cresol pathway by exerting beneficial effects on the diversity of gut microbiota.
(163)	Single-center, double-blind, randomized, placebo-controlled clinical trial	Chronic kidney disease (CKD) patients with diabetes and/or hypertension and at clinical stages 2, 3, and 4	Participants were randomly assigned to receive FMT or placebo in a 1:1 ratio for 6 months.	–	After fecal microbiota transplantation (FMT), the progression of chronic kidney disease (CKD) in patients is slowed. Oral administration of FMT capsules is a safe strategy for CKD patients, carrying no risks and offering potential benefits.

Crucially, the quality of donor microbiota determines therapeutic efficacy. Wang et al. found that transplanting microbiota from ESRD patients into germ-free CKD mice exacerbated uremic toxin accumulation, oxidative stress, and renal interstitial fibrosis (158), highlighting the importance of donor microbiota selection. Lu et al. showed that FMT from healthy donors restored podocyte insulin sensitivity and mitigated glomerular damage in diabetic rats with antibiotic-induced microbiota depletion (159), advancing mechanistic insights into microbiota-host metabolic interactions at the cellular level. Clinical evidence for FMT in renal diseases remains limited but includes notable case reports. Zhao et al. described two Chinese female IgA nephropathy patients who received intensive fresh FMT for 6–7 months, resulting in 24 h urinary protein (24 hUP) levels reduced to less than half of baseline, increased serum albumin (sAlb), stable renal function, and restored gut microbial balance (160). Zhou et al. reported a refractory membranous nephropathy patient who, after two FMT treatments, exhibited elevated serum albumin and total protein levels, reduced creatinine and 24 hUP, decreased anti-phospholipase A2 receptor (PLA2R) antibody titers, and resolution of edema and diarrhea (161). These cases suggest FMT may modulate autoimmune kidney diseases via the microbiota-immune axis. In dialysis-related infections, Marasco et al. successfully treated a peritoneal dialysis patient with recurrent Clostridioides difficile infection using colonoscopic FMT (162).

Despite its established role in infection control, FMT’s efficacy in ESRD/CKD populations lacks robust clinical validation. Current research focuses on animal models (e.g., diabetic nephropathy, TBI) and small pilot studies, with a complete absence of hard clinical endpoints such as improved eGFR or reduced cardiovascular events. The successful application of FMT in IgA nephropathy and membranous nephropathy may support further exploration of its therapeutic potential in ESRD. However, short-term adverse effects (diarrhea, abdominal pain, bloating, constipation, fever) and theoretical long-term risks (e.g., rheumatoid arthritis, irritable bowel syndrome) remain concerns (15). Thus, widespread adoption of FMT in dialysis populations requires bridging the gap between mechanistic plausibility and clinical evidence.

### 7.5 Emerging therapies in ESRD management

Novel therapeutic approaches are gaining attention for ESRD patients. For example:

AST-120: Developed in Japan, this porous carbon particle (0.2–0.4 mm diameter) adsorbs tryptophan metabolites (e.g., indole), reducing precursors for hepatic indoxyl sulfate synthesis and slowing renal deterioration (164). Wu et al. confirmed that AST-120 alleviates uremic pruritus severity in ESRD patients (165). A retrospective cohort study by Kweon et al. showed that pre-dialysis use of AST-120 for ≥ 4 months significantly reduced post-dialysis cardiovascular events and composite outcomes (166).

Sevelamer: A large cationic polymer that binds phosphate via protonated amine groups, forming non-absorbable complexes to lower serum phosphorus. Sevelamer also reduces endotoxin and CD14 levels in HD patients, potentially improving microinflammation and oxidative stress (167). Zeng Q et al. demonstrated in a randomized open-label trial that sevelamer delays vascular calcification, thereby reducing post-dialysis cardiovascular risks (168).

Although the diabetic population is not the primary focus of this review, it is noteworthy that a higher proportion of individuals with diabetes develop chronic kidney disease (CKD), with diabetic nephropathy being a leading cause of end-stage renal disease (169).

A recent breakthrough study revealed that diphenyl diselenide (DPDS) selectively increases the relative abundance of probiotics and enhances microbial diversity. It significantly improves the gut microbiota composition in streptozotocin (STZ)-induced type 1 diabetic rats, regulates intestinal microbial homeostasis, and ameliorates renal function in STZ-treated rats (170). This research demonstrates the potential application value of diphenyl diselenide in kidney diseases.

Beyond the above, gut microbiota modulation-based Chinese herbal medicines and plant-derived natural products have also demonstrated unique potential, offering multi-dimensional intervention strategies for end-stage renal disease (ESRD) management. In recent years, multiple clinical studies have confirmed that natural products represented by compound Chinese herbal formulas or active plant components can provide adjuvant therapy for ESRD patients by regulating gut microbiota dysbiosis, reducing uremic toxin production, and improving intestinal barrier function (171, 172).

For example:

Researchers have identified that mild-natured, sweet-flavored traditional Chinese medicines (TCMs) enrich *Lactobacillaceae* and *Bifidobacterium*, downregulate *Enterobacteriaceae*, and remodel short-chain fatty acid (SCFA) metabolism (173).

Concurrently, the herbal medicine *Clerodendranthus spicatus* (syn. *Orthosiphon aristatus*) enriches *Lachnospiraceae* and *Alloprevotella*, inhibits the URAT1 urate transporter, reduces serum uric acid levels, suppresses renal NLRP3 inflammasome activation, and restrains renal inflammation in hyperuricemic nephropathy (174).

Lin et al. conducted a randomized controlled trial investigating the effects of Fushen Granule (FSG) in patients with peritoneal dialysis-related peritonitis (PDRP). FSG enriched metabolism-related beneficial bacteria (e.g., Ruminococcus), significantly reduced blood urea nitrogen (BUN) and serum creatinine (Scr) levels in PDRP patients, increased albumin (ALB), and improved patients’ nutritional status (175). Plant-derived active components such as curcumin (a turmeric extract) have shown clear toxin clearance and anti-inflammatory effects in HD patients: 3 months curcumin supplementation significantly reduced serum p-cresyl sulfate (PCS) levels (176). Animal model studies have further corroborated the potential mechanisms of natural products: Ji’s research team confirmed in a 5/6 nephrectomy rat model that rhubarb enema treatment inhibited the overgrowth of conditionally pathogenic gut bacteria, including Akkermansia, Methanosphaera, and Clostridiaceae. Rhubarb enema improved the intestinal barrier, regulated gut microbiota dysbiosis, inhibited systemic inflammation, and alleviated renal fibrosis (177). Zhang et al. found that thonningianin A (TA) may exert protective effects against renal interstitial fibrosis in diabetic nephropathy rats by regulating gut microbiota dysbiosis, ameliorating intestinal mucosal barrier damage, reducing the production and release of LPS, inhibiting NLRP3/ASC/Caspase-1 signaling pathway activation, and suppressing renal inflammatory responses (178).

**FIGURE 2:**
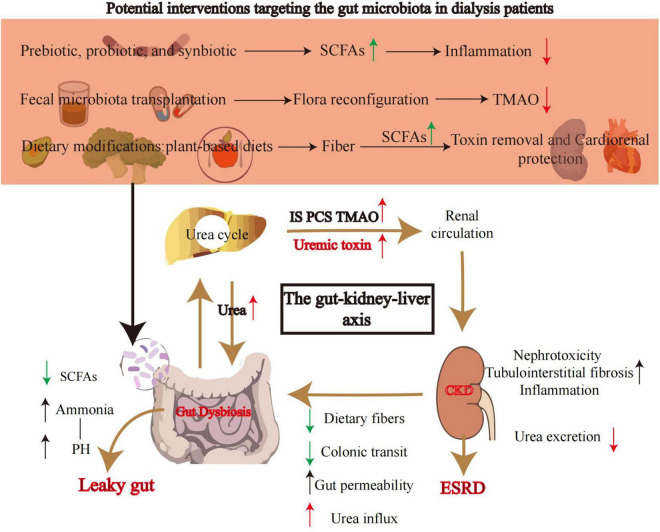
Core mechanisms of the gut-kidney axis and potential gut microbiota-targeted interventions. This diagram illustrates the core role of the gut microbiota in the progression of chronic kidney disease (CKD) to end-stage renal disease (ESRD) and the targeted gut microbiota intervention strategies. Prebiotics/probiotics/synbiotics: regulate microbial composition to promote the production of short-chain fatty acids (SCFAs). SCFAs enhance the intestinal mucosal barrier and suppress inflammatory factors (e.g., IL-6), reducing systemic microinflammation. Fecal microbiota transplantation (FMT): reconstructs the gut microbial ecosystem with healthy donor microbiota to inhibit TMAO-producing bacteria and reduce circulating TMAO levels. Plant-based diet (PBD): increases dietary fiber intake to promote SCFA production and reduce uremic toxin precursors, achieving dual benefits of “toxin clearance-cardiorenal protection.” Gut-kidney-liver axis mechanisms - urea and toxin accumulation: urea from hepatic metabolism enters the gut via renal circulation and is hydrolyzed into ammonia by bacterial urease (leading to increased gut pH). Uremic toxins [e.g., indoxyl sulfate (IS), p-cresyl sulfate (PCS), TMAO[accumulate due to reduced renal function. Intestinal leakage formation: in CKD patients, reduced dietary fiber, slowed colonic transit (constipation), and increased urea influx lead to decreased SCFA production and overgrowth of pathogenic bacteria (gut dysbiosis). Ammonia and toxins disrupt the intestinal mucosal barrier, increasing gut permeability (“intestinal leakage”) and exacerbating endotoxin translocation. Vicious cycle of renal injury: intestinal leakage causes endotoxin translocation into the circulatory system, triggering systemic inflammation (Inflammation). Circulating toxins (IS, PCS) induce tubulointerstitial fibrosis and nephrotoxic damage via the aryl hydrocarbon receptor (AhR) pathway. The interplay between inflammation and toxin accumulation ultimately accelerates the progression of CKD to ESRD. Arrow colors in the diagram - green arrows: positive regulation; red arrows: negative regulation. CKD, chronic kidney disease; ESRD, end-stage renal disease; SCFAs, short-chain fatty acids; IS, indoxyl sulfate; PCS, p-cresyl sulfate; TMAO, trimethylamine N-oxide.

In summary, these natural products and synthetic compounds, through multi-acting mechanisms such as “modulating microbiota-reducing toxins-repairing barriers,” synergize with traditional kidney replacement therapies (e.g., HD/PD), providing more personalized treatment options for ESRD patients (Figure 2).

## 8 Conclusion

Targeted interventions focusing on the gut microbiome offer a novel perspective for the precision management of end-stage renal disease (ESRD). Current research reveals that hemodialysis (HD) patients are characterized by enrichment of Firmicutes and Proteobacteria, while peritoneal dialysis (PD) patients exhibit reduced microbial diversity and increased pathogenic bacterial proliferation due to long-term exposure to high-glucose dialysate and iron supplementation. Probiotics, prebiotics, and plant-based diets demonstrate clinical potential by modulating short-chain fatty acid (SCFA) metabolism and suppressing pathogenic bacteria, though their efficacy is significantly influenced by host microbial profiles, intervention dosages, and kidney replacement therapy (KRT) modalities. Although fecal microbiota transplantation (FMT) has validated toxin clearance and metabolic improvements in animal models, its long-term safety and hard clinical endpoint benefits in immunocompromised ESRD populations still require large-scale clinical validation.

The core challenge in current research lies in the complexity of interactions among therapeutic interventions, host factors, and the microbiome. The dynamic impact of KRT on microbial communities has not been fully elucidated. Both HD and PD not only directly alter gut microbiota composition but may also trigger multi-organ pathological changes through mechanisms such as blood microbial translocation (HD) or peritoneal microbiome imbalance (PD). Future studies must adopt standardized microbial strain selection, larger sample sizes, and extended follow-up periods to clarify the clinical impact of prebiotics, probiotics, and synbiotics in ESRD patients. Simultaneously, it is imperative to reassess the rationale behind traditional dietary restrictions and explore synergistic effects between plant-based diets and microbiome-targeted therapies. In the era of personalized medicine, microbiota-specific research will undoubtedly occupy a central position. Overcoming the gap between mechanistic understanding and clinical translation will pave the way for individualized KRT management strategies, ultimately bringing profound benefits to ESRD patients.
